# Perioperative Takotsubo Cardiomyopathy Revealed by Ventricular Arrhythmia After a Minor Surgery in a Young Woman

**DOI:** 10.7759/cureus.101699

**Published:** 2026-01-16

**Authors:** Sara Hafid, Samy Lebbar, Ghita Bennis, Soukaina Scadi, Fatimazahra Merzouk, Ghali Benouna

**Affiliations:** 1 Cardiology, Cheikh Khalifa International University Hospital, Mohammed VI University of Health Sciences, Casablanca, MAR; 2 Cardiology, Mohammed VI International University Hospital, Casablanca, MAR; 3 Cardiology, Mohammed VI International University Hospital, Mohammed VI University of Health Sciences, Casablanca, MAR

**Keywords:** cardiac mri, general anesthesia, stapedotomy, takotsubo cardiomyopathy (ttc), ventricular tachycardia (vt)

## Abstract

Takotsubo syndrome (TTS) is an acute, reversible form of stress-induced cardiomyopathy typically triggered by intense emotional or physical stress. Perioperative presentations are uncommon but increasingly recognized, and may present abruptly with severe hemodynamic instability or malignant arrhythmias. These forms can mimic anesthetic complications or acute coronary syndromes, delaying diagnosis. We report the case of a healthy 32-year-old woman who developed sustained monomorphic ventricular tachycardia followed by cardiac arrest 15 minutes after an uncomplicated stapedotomy under general anesthesia. Return of spontaneous circulation was achieved after two synchronized shocks. Post-resuscitation, vasoactive support was initiated after bedside echocardiography revealed a markedly reduced left ventricular ejection fraction (≈25%) with apical ballooning, consistent with Takotsubo cardiomyopathy. The post-arrest ECG revealed lateral T-wave inversions with QTc prolongation to 480 ms. Biomarkers showed a modest rise in high-sensitivity troponin. Left ventricular function partially improved within 48 hours and fully normalized at one week. Cardiac MRI confirmed diffuse myocardial edema without late gadolinium enhancement, consistent with Takotsubo cardiomyopathy. This case illustrates a rare but severe perioperative presentation of TTS, manifesting as ventricular tachyarrhythmia and cardiac arrest in a young patient undergoing minor otologic surgery. It highlights the importance of considering TTS in cases of unexplained intraoperative or immediate postoperative cardiovascular collapse, and underscores the diagnostic value of multimodality imaging, particularly cardiac MRI, in distinguishing TTS from ischemic myocardial injury.

## Introduction

Takotsubo syndrome (TTS) is characterized by an acute and reversible systolic dysfunction of the left ventricle, typically triggered by intense emotional or physical stress, and defined by transient regional wall motion abnormalities most often involving the apical segments, in the absence of obstructive coronary artery disease [1].

Although initially described in postmenopausal women, perioperative forms are increasingly recognized in the anesthesiology and surgical literature [2-4]. These variants often present atypically, lack prodromal chest pain, and occur in the immediate postoperative period, sometimes with severe hemodynamic instability, including acute hypotension or cardiogenic shock, and malignant arrhythmias such as ventricular tachycardia or fibrillation [5].

Surgical stress, anesthetic agents, intubation and extubation stimuli, abrupt hemodynamic changes, pain, and excessive catecholamine surges, leading to autonomic imbalance and myocardial stress, form a constellation of potent physiological triggers capable of precipitating stress-induced cardiomyopathy [4,6,7].

The pathophysiology of TTS is not fully understood but is thought to involve catecholamine-mediated myocardial injury through direct cardiotoxic effects, microvascular dysfunction, and transient myocardial stunning, resulting in characteristic regional ventricular dysfunction. The perioperative form appears to carry a higher risk of complications than classical TTS, including cardiogenic shock, arrhythmias, and cardiac arrest, and may develop during anesthesia induction, intraoperatively, or in the early postoperative phase [4,6,8]. In this context, prompt recognition is essential, as the presentation may mimic acute coronary syndrome or anesthetic complications, and delayed diagnosis can lead to inappropriate management.

This report describes a rare and severe case of perioperative TTS in a young woman undergoing minor otologic surgery, presenting initially with recurrent ventricular tachycardia and cardiac arrest, an uncommon presentation, as perioperative TTS accounts for fewer than 10% of reported cases and ventricular arrhythmias occur in only approximately 2.7-3.4% of patients, likely related to acute anesthesia- and surgery-induced sympathetic activation, catecholamine excess, and transient myocardial electrical instability, and aims to highlight the pathophysiology, clinical features, and implications of perioperative stress-induced cardiomyopathy within the context of existing literature [2,4,8].

## Case presentation

A 32-year-old woman with no prior cardiovascular disease was admitted for elective stapedotomy under general anesthesia. Her medical history was unremarkable, with no cardiovascular risk factors, psychiatric disorders, or recent emotional or physical stress. She had previously undergone two cesarean sections and a laparotomy for endometriosis, all without perioperative complications. Preoperative assessment showed normal vital signs and a normal 12-lead ECG.

**Figure 1:**
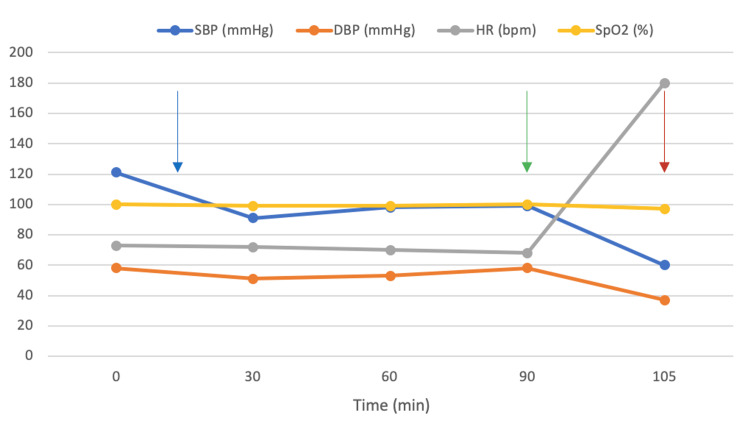
Intraoperative hemodynamic monitoring during general anesthesia. Blue arrow: Anesthesia induction and orotracheal intubation. Green arrow: End of the surgical procedure. Red arrow: Onset of ventricular tachycardia. SBP = systolic blood pressure; DBP = diastolic blood pressure; HR=  heart rate; SpO₂ = oxygen saturation

Anesthesia was induced with midazolam (2 mg), ketamine (30 mg), and propofol (200 mg, followed by 50-mg boluses every 30 minutes), combined with fentanyl (200 µg) and rocuronium (40 mg, with a 10-mg supplement after 30 minutes). Maintenance was achieved with sevoflurane. Prophylactic intravenous cefazolin and ondansetron were administered. Intraoperative hemodynamics remained relatively stable. A transient decrease in systolic blood pressure to 91-99 mmHg was managed with intravenous isotonic saline infusion, after which systolic blood pressure remained stable around 100 mmHg without the need for vasopressor support. Heart rate ranged between 70 and 73 beats per minute, and oxygen saturation was maintained at 99%. No arrhythmias or ischemic changes were noted during the procedure. At the end of surgery, the patient remained intubated while awaiting full emergence from anesthesia and completion of the surgical dressing (Figure 1).

Approximately 15 minutes after the end of the procedure, during the emergence phase of anesthesia and while still intubated, the patient developed sudden hemodynamic instability followed by a sustained monomorphic ventricular tachycardia documented on continuous cardiac monitoring. The first episode was a ventricular tachycardia with preserved pulse and was treated with immediate synchronized electrical cardioversion at 150 J, resulting in transient restoration of sinus rhythm. Shortly thereafter, a second episode of ventricular tachycardia occurred and rapidly degenerated into pulseless ventricular tachycardia, requiring brief cardiopulmonary resuscitation and immediate defibrillation at 200 J, which successfully restored a stable sinus rhythm. The patient did not experience laryngospasm, coughing, agitation, or any airway complications. Endotracheal intubation was maintained for airway protection following the cardiac arrest.

Immediately after cardioversion, vasoactive support with epinephrine and norepinephrine was initiated. A bedside transthoracic echocardiogram performed by the intensive care team within minutes revealed severe left ventricular systolic dysfunction with an estimated left ventricular ejection fraction (LVEF) of approximately 20%. In this context, dobutamine was introduced for inotropic support. Epinephrine was initially administered as part of post-resuscitation management and subsequently continued as a continuous infusion. Following early cardiology consultation and the suspicion of TTS, catecholaminergic support was rapidly de-escalated, with progressive weaning of norepinephrine, epinephrine, and dobutamine as hemodynamic status improved (Table 1). This strategy aimed to balance immediate hemodynamic stabilization with the recognition that sustained catecholaminergic stimulation may exacerbate stress-induced cardiomyopathy. Peri-arrest laboratory evaluation, including serum electrolytes and arterial blood gas analysis, showed no significant metabolic, electrolyte, or acid-base abnormalities (Table 2).

**Table 1:** Hemodynamic monitoring and medication timeline. *: Twice a day. Pre-op = preoperative; Post-op = postoperative; LVEF = left ventricular ejection fraction

	Pre-op	Post-op 0 hour	4 hours post-arrest	24 hours post-arrest	72 hours post-arrest	One week post-arrest	Normal range
Systolic blood pressure (mmHg)	121	60	116	111	118	120	90–130
Diastolic blood pressure (mmHg)	58	40	95	67	72	65	60–80
Heart rate (beats/minute)	73	122	120	126	75	68	60–100
LVEF (%)	-	20%	23%	-	35%	65%	>50%
Epinephrine (mg/kg/minute)	-	0.42	0.21	0	0	0	-
Norepinephrine (mg/kg/minute)	-	1.2	0	0	0	0	-
Dobutamine (µg/kg/minute)	-	8.3	4.2	0	0	0	-
Carvedilol (mg)*	0	0	0	3.125	6.25	6.25	-

**Table 2:** Timeline of laboratory parameters. *: Blood samples and arterial blood gas were obtained immediately after resuscitation. Pre-op = preoperative; Post-op = postoperative; CRP = C-reactive protein

	Pre-op	Post-op 0 hour*	24 hours post-arrest	72 hours post-arrest	Normal range
Hemoglobin (g/dL)	13.7	13.2	-	12.9	12–16
White blood cells/mm^3^	8,700	9,500	-	9,600	4,000–10,000
Platelets/mm^3^	280,000	276,000	-	252,000	150,000–400,000
CRP (mg/L)	-	8	-	10	<5
Na+ mmol/L	143	141	-	137	135–145
K+ (mmol/L)	3.8	3.5	-	4.0	3.5–5
Creatinine (mg/L)	10.6	11.8	-	8.8	7–13
Troponin I (ng/mL)	-	0.7	0.174	-	<0.03
PH	-	7.468	-	-	7.35–7.45
PCO_2_ (mmHg)	-	36.2	-	-	35–45
PO_2_ (mmHg)	-	92	-	-	80–100
HCO_3_^-^ (mmol/L)	-	26.2	-	-	22–26

Following hemodynamic stabilization, the patient was successfully extubated four hours later in the intensive care unit. On awakening, the patient denied significant pain on clinical assessment, and no signs of inadequate postoperative analgesia or agitation were noted. A comprehensive transthoracic echocardiogram performed three hours after the event confirmed severe left ventricular dysfunction with marked apical akinesia and basal hyperkinesis, consistent with apical ballooning, and an LVEF of 23% by the Simpson biplane method. There was no left ventricular outflow tract obstruction or apical thrombus. Right ventricular size and function were normal, and no pericardial effusion was observed.

Post-arrest electrocardiography showed sinus tachycardia at 125 beats per minute, lateral T-wave inversions, and a prolonged corrected QT interval (QTc) of 480 ms. High-sensitivity troponin levels were mildly elevated, peaking at 0.70 ng/mL before declining over the following 24 hours, a pattern considered disproportionate to the severity of left ventricular dysfunction. Inflammatory markers remained low, and renal function was preserved (Table 2). After 24 hours of dobutamine washout, beta-blocker therapy with carvedilol was initiated at a dose of 3.125 mg twice daily.

Follow-up echocardiography at 48 hours demonstrated partial recovery of systolic function with an LVEF of 35%. Coronary angiography was deferred given the absence of chest pain, lack of ST-segment elevation, modest troponin elevation, and rapid improvement in left ventricular function, making an acute coronary syndrome unlikely; this decision was further supported by the patient’s informed refusal of invasive coronary angiography.

**Figure 2:**
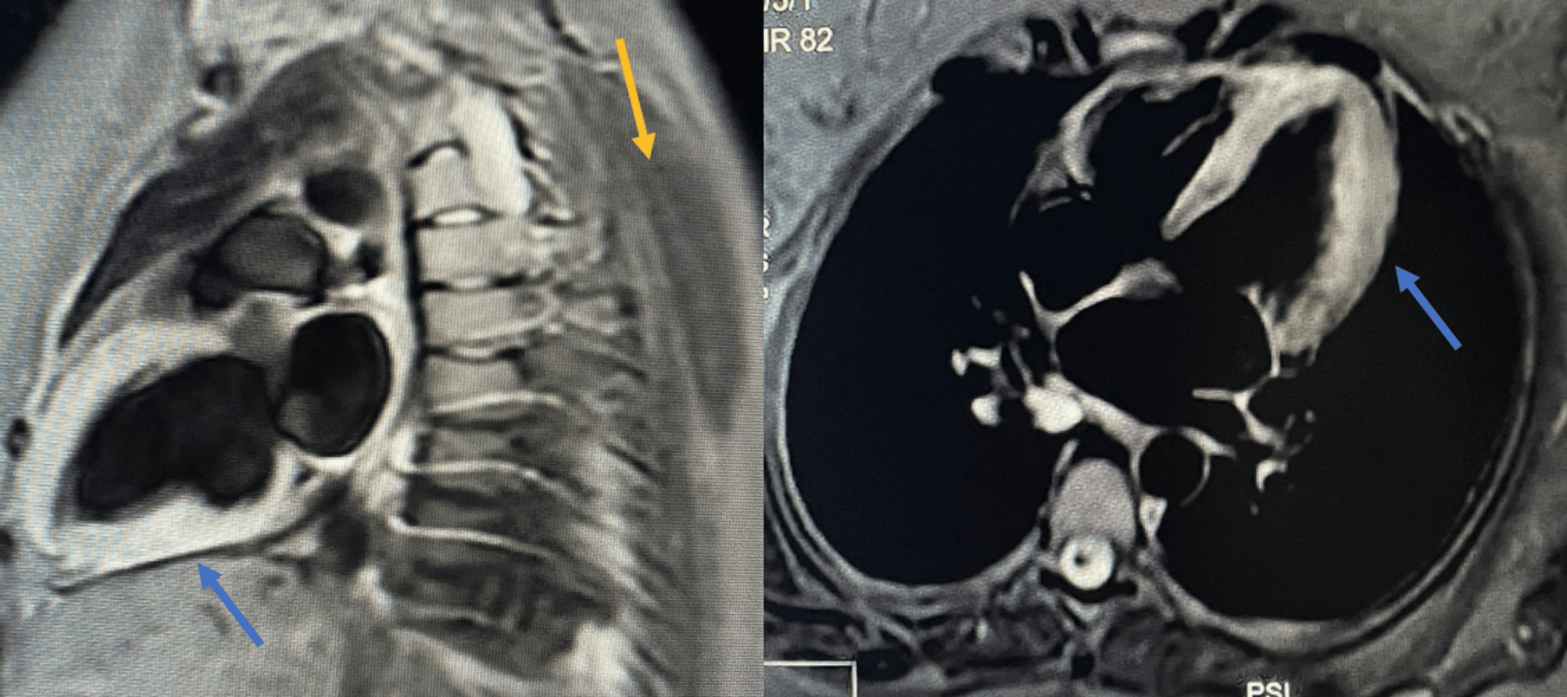
Cardiac MRI T2 short tau inversion recovery sequence in the two-chamber (left) and in the four-chamber (right) view: diffuse myocardial T2 hyperintensity compatible with myocardial edema, in comparison with adjacent dorsal skeletal muscles. Blue arrow: Myocardial hyperintensity. Yellow arrow: Dorsal skeletel muscles.

**Figure 3:**
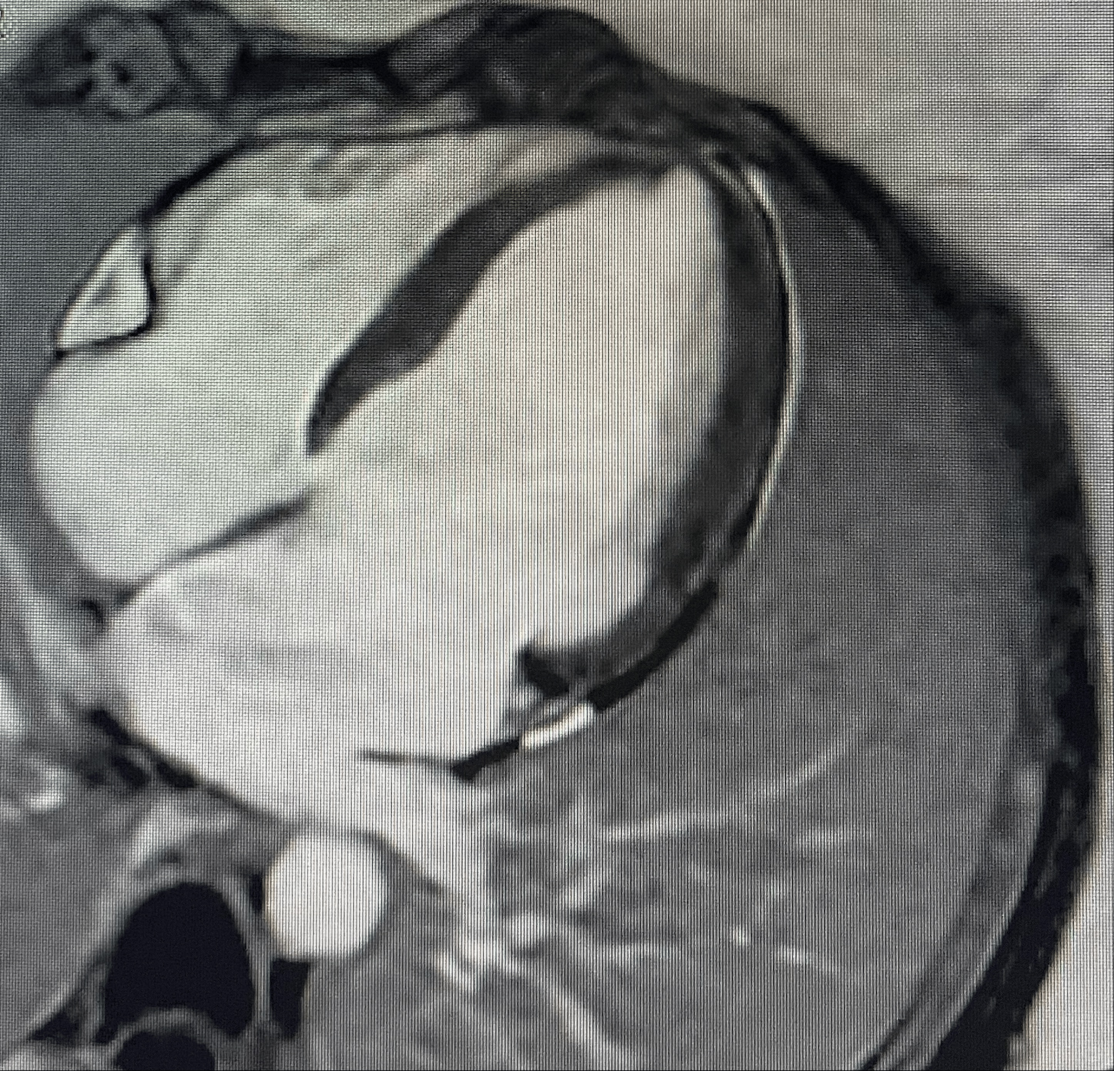
Late gadolinium enhancement sequence: no late gadolinium enhancement is observed.

One week later, a cardiac MRI was performed. It demonstrated diffuse, moderate myocardial edema on T2-short tau inversion recovery sequences (Figure 2), elevated T2 mapping values in the lateral wall (68-73 ms) and septum (~54 ms), with no late gadolinium enhancement (Figure 3). Left ventricular volumes and mass were normal; systolic function had normalized (LVEF: 67%, right ventricular ejection fraction: 61%). These findings were fully consistent with Takotsubo cardiomyopathy.

Follow-up transthoracic echocardiography confirmed full recovery of left ventricular function (LVEF: 65%). Both the QTc interval and T-wave abnormalities had normalized before hospital discharge. The patient remained asymptomatic, with no recurrence of arrhythmia, and was discharged on low-dose carvedilol (6.25 mg, twice a day), which she continued for three months before being lost to follow-up. Oral consent was obtained for publication.

## Discussion

The present case illustrates a severe perioperative presentation of TTS in a young, previously healthy patient who developed malignant ventricular tachyarrhythmia and cardiac arrest shortly after a low-risk procedure. Perioperative TTS is increasingly recognized as a distinct clinical entity and may occur during induction, intraoperatively, or in the early postoperative phase [4,8]. Although perioperative TTS represents a minority of Takotsubo presentations, it is rare (incidence ~0.016% (~1 in 6,400) after non-cardiac surgery, in a large retrospective cohort [8]) and may be associated with meaningful in-hospital mortality (10%) [8]. Perioperative cohorts suggest a higher burden of acute complications compared with non-perioperative TTS, including cardiogenic shock, life-threatening arrhythmias, and cardiac arrest [4,8]. Ventricular tachyarrhythmia as the initial manifestation of TTS is uncommon [2] and should be considered a marker of acute disease severity, reflecting profound sympathetic activation and transient myocardial electrical instability during the acute phase [5,8,9].

Several mechanisms may explain the susceptibility of the perioperative period to TTS. Physiological stressors and abrupt autonomic shifts related to surgery and anesthesia can trigger acute catecholamine surges, resulting in direct myocardial toxicity, microvascular dysfunction, and transient myocardial stunning [1,5,10]. In addition, perioperative hemodynamic changes and exposure to vasoactive agents may further contribute to adrenergic excess and myocardial vulnerability [4,5]. In our case, the event occurred during emergence from anesthesia, a period characterized by marked autonomic fluctuations, even in the absence of airway complications.

Multiple anesthetic agents have been implicated as potential contributors to perioperative autonomic imbalance. Propofol has been associated with rare peri-induction ventricular arrhythmias and paradoxical sympathetic activation [3,9,11], while ketamine increases circulating catecholamines and myocardial oxygen demand [10]. Opioids such as fentanyl may induce abrupt autonomic shifts, and volatile agents, including sevoflurane, can transiently depress myocardial contractility [4]. Although these drugs are routinely used and generally safe, their combined physiological effects may amplify perioperative sympathetic activation and potentially precipitate TTS in susceptible individuals. Similar cases have reported TTS presenting with malignant ventricular arrhythmias during induction or early recovery, reinforcing that severe episodes may follow even minor procedures [3,6,7,12].

Differential diagnoses in the perioperative setting include acute coronary syndrome, coronary vasospasm, myocarditis, and anesthetic-related cardiotoxicity. In the present case, acute coronary syndrome was considered unlikely due to the absence of chest pain, lack of ST-segment elevation, a modest troponin rise disproportionate to the degree of left ventricular dysfunction, and rapid functional recovery [2,5]. Myocarditis was effectively excluded by cardiac MRI, which demonstrated myocardial edema without late gadolinium enhancement, supporting TTS and arguing against infarction or inflammatory cardiomyopathy [5,13]. Although coronary angiography is generally recommended in suspected TTS, a non-invasive diagnostic strategy was favored in this young, low-risk patient, with early CMR confirmation providing a reliable alternative [5,13]. This approach is consistent with contemporary expert consensus recommendations for TTS [5].

From a management standpoint, severe perioperative TTS may require immediate vasoactive and inotropic support in the post-arrest setting. However, sustained catecholaminergic stimulation may exacerbate TTS pathophysiology; therefore, the lowest effective doses should be used and de-escalated as soon as clinically feasible once hemodynamic stabilization is achieved [5,10]. In cases of refractory cardiogenic shock with escalating catecholamine requirements, temporary mechanical circulatory support may be considered a bridge to recovery in selected patients [5].

Practical take-home messages for anesthesiologists and perioperative teams include early recognition of bedside red flags such as unexplained ventricular tachyarrhythmia and acute severe left ventricular dysfunction during induction, emergence, or early recovery, prompting urgent point-of-care echocardiography and multidisciplinary evaluation [4,5]. Perioperative management should aim to minimize adrenergic surges, avoid tachycardia when possible, and maintain stable preload and afterload. Multimodal analgesia should be optimized to prevent pain-related sympathetic activation. When vasopressor therapy is required, non-β-adrenergic agents such as phenylephrine or vasopressin may be preferred over β-agonists, while catecholamine inotropes should be reserved for severe low-output states and tapered early [4,5]. Following severe presentations, postoperative high-dependency or intensive care monitoring is advisable [4,8]. While this single case does not suggest a change in current perioperative risk stratification, it highlights that severe TTS can occur even after low-risk procedures and supports close monitoring in the immediate postoperative period when malignant arrhythmias or hemodynamic instability may occur.

Regarding follow-up, beta-blocker therapy is commonly continued for several weeks to months and reassessed after recovery of ventricular function [5]. Although long-term recurrence risk is generally low, it is not negligible; patients presenting with malignant arrhythmias during the acute phase may benefit from short-term rhythm surveillance (telemetry during hospitalization and ambulatory monitoring when clinically indicated), particularly in the presence of QT prolongation or recurrent symptoms [5].

## Conclusions

Perioperative Takotsubo cardiomyopathy remains an uncommon but potentially life-threatening complication, capable of presenting abruptly with malignant ventricular arrhythmias or hemodynamic collapse. This case highlights the importance of considering Takotsubo cardiomyopathy even in young patients with no cardiovascular risk factors undergoing seemingly minor surgery under general anesthesia and the need for heightened vigilance and close monitoring during anesthesia induction, emergence, and the immediate postoperative period. Early bedside echocardiography, prompt hemodynamic stabilization, and the judicious use of multimodality imaging, particularly cardiac MRI, are essential for establishing the diagnosis and avoiding unnecessary invasive procedures. Clinicians should maintain a high index of suspicion for stress-induced cardiomyopathy when faced with unexplained arrhythmias or acute ventricular dysfunction in the perioperative period, as timely recognition allows appropriate management and supports the excellent prognosis associated with this reversible condition.
